# Higher Animal Protein Intake During the Second Trimester of Pregnancy Is Associated With Risk of GDM

**DOI:** 10.3389/fnut.2021.718792

**Published:** 2021-11-15

**Authors:** Heng Yaw Yong, Zalilah Mohd Shariff, Barakatun Nisak Mohd Yusof, Zulida Rejali, Yvonne Yee Siang Tee, Jacques Bindels, Eline M. van der Beek

**Affiliations:** ^1^Department of Nutrition, Faculty of Medicine and Health Sciences, Universiti Putra Malaysia, Serdang, Malaysia; ^2^Department of Dietetics, Faculty of Medicine and Health Sciences, Universiti Putra Malaysia, Serdang, Malaysia; ^3^Department of Obstetrics and Gynaecology, Faculty of Medicine and Health Sciences, Universiti Putra Malaysia, Serdang, Malaysia; ^4^Danone Specialized Nutrition (Malaysia) Sdn. Bhd., Kuala Lumpur, Malaysia; ^5^Nutricia Research Foundation, Nieuwvee, Netherlands; ^6^Department of Pediatrics, University Medical Centre Groningen, University of Groningen, Groningen, Netherlands

**Keywords:** egg protein, animal protein, red meat protein, gestational diabetes (GDM), before pregnancy, during pregnancy

## Abstract

**Background and Aims:** This study aimed to examine the associations between the total protein intake as well as types and sources of proteins with the gestational diabetes mellitus (GDM) risk.

**Method and Results:** This was a prospective cohort study of the pregnant women in Malaysia. In this study, the total, animal, and plant protein intakes were assessed using a semi-quantitative food frequency questionnaire. Of the 452 women, 48 (10.62%) were diagnosed with GDM. From pre-pregnancy to second trimester, most of the women had 10–20% of energy intake from protein (88.9–90.3%) and ≥75% of recommended protein intake (74.6–86.5%). The women in the highest tertile (T3) of total animal protein intake [adjusted odds ratio (AOR) = 2.76, 95% *CI* = 1.27–6.04] and red meat protein (AOR = 2.69, 95% *CI* = 1.27–5.70), specifically in the second trimester, had significantly higher GDM risk compared with the women in the middle tertile of intake (T2). Interestingly, the women in the T3 of egg protein in the second trimester were significantly at lower GDM risk (AOR = 0.43, 95% *CI* = 0.18–0.91) compared with those in T2.

**Conclusion:** The highest tertile of animal protein (≥42.15 g/day) intake, particularly red meat protein in the second trimester was positively associated with the GDM risk, whereas the highest tertile of egg protein was inversely associated with the GDM risk. Protein intake before or during early pregnancy was not associated with the GDM risk. These findings underscore the importance of sources and types of protein intake, particularly after the first trimester of pregnancy, in relation to GDM risk.

## Introduction

The previous studies have focused on macronutrient intake in relation to the non-communicable diseases (NCDs), such as diabetes mellitus (DM), cardiovascular disease (CVD), chronic respiratory diseases, and cancer but mostly on the possible associations with carbohydrate and fat content in the diet (1–3). Recent evidence has shown that protein intake, both quantity and quality, might play an important role in metabolic health and NCDs (4). High protein intake is associated with the higher rates of NCDs, the current leading causes of morbidity and mortality (5, 6) globally. Adequate dietary protein during pregnancy is important to ensure the healthy pregnancy outcomes (7). Protein requirements increase over the course of pregnancy, yet the effects of high protein intake on the pregnancy outcomes, such as risk for gestational diabetes mellitus (GDM), excessive gestational weight gain (GWG), and hypertension are less well documented (8, 9).

The animal and plant proteins may have different effects on the metabolic disorders, such as DM and obesity (5, 10). Red and/or processed meat, major sources of animal protein that are often considered as unhealthy due to the cholesterol, saturated fat, and sodium contents (11), have been shown to be associated with the risk of type 2 diabetes mellitus (T2DM) (12, 13) and GDM (14, 15). While there is increasing evidence to support the inverse relationship between the dairy products, particularly those of low-fat, with risk of obesity, T2DM, and GDM (16–18), and the associations between the other major sources of animal protein (e.g., poultry, fish, and dairy products) with the risks of T2DM and GDM have not been well reported. On the other hand, the higher intakes of plant-protein specifically nuts and legumes are associated with a lower risk of T2DM and GDM (19, 20).

Globally, dietary protein intake has increased substantially during the last few decades (21). As a result of urbanization and nutrition transition, meat consumption in Malaysia has continued to rise over the past century with the largest proportion from red meat (58%) (22). The annual per capita consumption of beef and pork in Malaysia has increased from 1.6 and 2.3 kg in 1960 to 5.7 and 17.9 kg in 2018 (23, 24). Over the same period, the prevalence of obesity in the women of childbearing age has risen dramatically, whereby in 2019, about 24.7% women of childbearing age were obese (25). Combinedly these factors may contribute to an increased risk of hyperglycemia during pregnancy. As an existing evidence on protein intake and GDM risk is mainly based on the Western population (9, 14), it is worthwhile to investigate such association in the Asian population that is undergoing rapid socio-economic and nutrition transitions. This study aimed to examine the associations between the total protein intake as well as the types (animal vs. plant) and sources of plant and animal proteins with the risk of GDM.

## Methods

### Study Design

The Seremban Cohort Study (SECOST) project was a prospective cohort study in which the pregnant women were followed-up to 1 year postpartum, and their infants were followed-up every 6 months until 2-year old. The women in the first trimester (<10th week of gestation) of pregnancy were recruited from the three maternal and child health (MCH) clinics in Seremban District, Negeri Sembilan, Malaysia. Detailed descriptions of the study methodology are previously published (26). All the pregnant women were eligible to participate unless they had one or more exclusion criteria as published previously. A total of 737 pregnant women were enrolled in the SECOST study but 22.7% (*n* = 167) and 16.0% (*n* = 118) were excluded at first and second visit, respectively. The present study reported on data of 452 pregnant women only.

The study protocol was approved by the Medical Research Ethics Committee (MREC), Universiti Putra Malaysia (UPM/FPSK/100-9/2-MJKEtika), and the Medical Research Ethics Committee (MREC), the Ministry of Health Malaysia (KKM/NIHSEC/08/0804/P12-613). The permission to conduct this study was obtained from the Head of Seremban District Health Office. All the women gave written informed consent before study enrollment.

## Measurements

### Dietary Assessment

Dietary intake of the women was assessed using a 126-food item semi-quantitative frequency questionnaire (SFFQ), which validated specifically to assess dietary intake among the Malaysian (27). The women were asked to designate the frequency and portion size of their intake of each food item in that particular trimester, namely, the first prenatal visit (9.82 ± 2.51 weeks of gestation to collect data on dietary intake before pregnancy), the first trimester (12.26 ± 1.58 weeks of gestation) and the second trimester (26.73 ± 1.64 weeks of gestation), respectively. The consumption of each food item was then converted into daily food item intake (g/day). The dietary data were analyzed using Nutritionist Pro Diet Analysis software: Version 1.5 (28) with the United States Department of Agriculture (USDA) food database (29). Each food item was categorized as either plant protein or animal protein. The protein sources from either plant protein or animal protein were categorized into 12 food groups (Supplementary Table 1). Protein intake for each food group was calculated by summing the protein content (g/day) of food items in the respective food group. Total protein intake (g/day) was derived as the sum of animal and plant protein. The medians of total protein intake before and during pregnancy were compared with the Recommended Nutrient Intakes (RNI) for Malaysia (30). Each type of protein intake was further divided into tertiles (T1, T2, and T3) using the visual binning tool in SPSS. The T2 was set as the reference group in the multivariate logistic regression model. For the tertiles of total protein intake before pregnancy and each trimester, the T1 was < 80–90% of RNI, T2 was between 90 and 120% of RNI, and T3 was more than 130% of RNI.

Implausible reporting was calculated using the Goldberg method (rEI:BMR) with reporters had rEI:BMR values that differed from the physical activity levels by more than ± 2 SDs (31, 32). The percentages of implausible reporting among the women at pre-pregnancy and during the first and second trimesters were 40.2, 51.2, and 50.3%, respectively. Despite the implausible reporters, the energy intake estimates was within the range of ± 3 SDs from the mean (33).

### Gestational Diabetes Mellitus

A two-point diagnostic 75 g oral glucose tolerance test (OGTT) was performed at 28–32nd weeks of gestation. A 2-ml fasting venous blood was drawn by a clinic staff nurse before ingestion of a standard glucose solution to obtain fasting plasma glucose (FPG). Another 2 ml of venous blood was drawn at 2-h after ingestion of standard glucose solution. All the blood samples were sent for analysis on the same day to determine FPG and 2-h plasma glucose (2hPG) concentration. GDM was diagnosed if either or both FPG ≥ 5.6 mmol/L or 2hPG ≥ 7.8 mmol/L according to the Ministry of Health (MoH) Malaysia guideline (34).

### Other Variables

Socio-demographic information, such as age, ethnicity, education level, occupation status, and monthly household income. Obstetrical information (e.g., parity, medical history GDM, and family history of DM) was obtained from the antenatal clinic cards. Height was measured at study enrollment, while weight was measured at each study visit using a standard instrument (SECA digital weighing scale and SECA body meter, CA, USA) and standard procedures. The women were requested to recall pre-pregnancy body weight. Pre-pregnancy body mass index (BMI) (kg/m^2^) was calculated as pre-pregnancy weight divided by the square of height and classified according to the recommendation of the WHO (35). Rate of weight gain in the second trimester was defined as the average weekly weight gain in the second trimester and was then classified based on the Institute of Medicine (IOM) recommendations (36).

### Statistical Analysis

All the analyses were performed using SPSS version 24 (IBM, NY, USA). An exploratory data analysis (EDA) was carried out to determine the normality and homogeneity of the data. The basic descriptive statistics were generated, such as means and SDs for normally distributed continuous variables, while median and percentiles (25 and 75th) for the non-normally distributed continuous variables. The categorical variables were reported as frequency with percentage. Kruskal–Wallis tests were performed to examine the differences in protein intake before and during pregnancy. A logistic regression was performed to assess the association between type of protein intake (animal vs. plant) and the sources of protein intake (food groups of animal protein vs. food groups of plant protein) with the risk of GDM, adjusted with covariates. The covariates included in the multivariable model were age, parity, family history of DM, history of GDM, pre-pregnancy BMI, energy intake, and fat intake. The associations between the sources of animal protein and GDM risk are reported as only animal protein intake showed significant association with the risk of GDM. The adjusted odds ratio (AOR) with 95% *CI* are presented.

## Results

The sample consisted of 88.9% Malay, and 11.1% non-Malay (Table 1). The mean age of women was 30.01 ± 4.48 years with 60.4% were 30 years old and below. Most of the women completed secondary education or lower (46.0%) and were employed (68.6%), from low-income households (62.8%) and nulliparous (35.4%). Only 6.9% of women had medical history of GDM and 24.6% had family history of DM. The mean height and pre-pregnancy body weight were 1.56 ± 0.06 m and 58.14 ± 12.92 kg. More than half (55.3%) had normal pre-pregnancy BMI, 34.1% were overweight or obese before pregnancy. The mean rate of GWG was 0.38 ± 0.12 kg/week, with the women were almost equally distributed among the three groups: inadequate (33.6%), adequate (32.8%), and excessive (33.6%). The mean daily energy intake for pre-pregnancy, first trimester, and second trimester of pregnancy were 2,159 ± 980.17 kcal/day, 2,033 ± 913.87 kcal/day, and 2,172 ± 896.90 kcal/day, respectively. The women had adequate protein intake before and during pregnancy with most women had 10–20% of energy from protein (88.9–90.3%) and ≥ 75% of RNI for protein (74.6–86.5%). About 10.6% were diagnosed with GDM.

**Table 1:** The characteristics of the women (*n* = 452).

**Characteristics**	***n*** **(%)**	**Mean ± SD**
Age at study entry (years)		30.01 ± 4.48
≤ 30	273 (60.4)	
> 30	179 (39.6)	
Ethnicity		
Malay	402 (88.9)	
Non-Malay	50 (11.1)	
Education level		12.96 ± 2.39
Secondary and lower	208 (46.0)	
STPM/Matric/Diploma/Certificate	148 (32.7)	
Tertiary and above	96 (21.3)	
Occupation		
Housewife	142 (31.4)	
Working	310 (68.6)	
Household income (RM)^a^		3726.74 ± 2050.96
Low (<3,860)	284 (62.8)	
Middle (3,860–8,319)	154 (34.1)	
High (≥8,320)	14 (3.1)	
Obstetrical information		
Parity		1.22 ± 0.45
0	160 (35.4)	
1–2	135 (29.9)	
≥3	157 (34.7)	
History of GDM		
No	421 (93.1)	
Yes	31 (6.9)	
Family history of DM		
No	341 (75.4)	
Yes	111 (24.6)	
Anthropometric measurements		
Height (m)		1.56 ± 0.06
Pre-pregnancy weight (kg)		58.14 ± 12.92
Pre-pregnancy BMI (kg/m^2^)		23.73 ± 4.80
Underweight (<18.5)	48 (10.6)	
Normal (18.5–24.9)	250 (55.3)	
Overweight (25.0–29.9)	103 (22.8)	
Obese (≥30.0)	51 (11.3)	
Rate of GWG (GWG) (kg/week)		0.38 ± 0.12
Second trimester		
Inadequate	152 (33.6)	
Adequate	148 (32.8)	
Excessive	152 (33.6)	
Energy and protein intake		
Total energy intake (kcal/day)		
Pre-pregnancy		2159 ± 980.17
First trimester		2033 ± 913.87
Second trimester		2172 ± 896.90
Protein intake (g/day)^b^		
Pre-pregnancy		59.43 (48.02, 78.22)
First trimester		55.84 (42.30, 76.17)
Second trimester		59.30 (45.04, 76.17)
Percentage of energy from protein (%)^c^		
Pre-pregnancy		14.52 ± 3.21
<10	20 (4.4)	
10–20	408 (90.3)	
>20	24 (5.3)	
First trimester		14.81 ± 3.08
<10	19 (4.2)	
10–20	402 (88.9)	
>20	31 (6.9)	
Second trimester		13.95 ± 2.74
<10	31 (6.9)	
10–20	408 (90.2)	
>20	13 (2.9)	
Percentage of RNI for protein intake (%)^b,d^		
Pre-pregnancy		112.88 (91.40, 148.96)
<75%	61 (13.5)	
75–100%	100 (22.1)	
>100%	291 (64.4)	
First trimester		106.04 (79.62, 136.69)
<75%	91 (20.1)	
75–100%	107 (23.7)	
>100%	254 (56.2)	
Second trimester		97.67 (74.29, 126.02)
<75%	115 (25.4)	
75–100%	118 (26.1)	
>100%	219 (48.5)	
Maternal glucose level		
Oral glucose tolerance test (OGTT)		
Gestational weeks at OGTT		28.00 ± 0.24
performed		
Fasting plasma glucose (FPG)		4.36 ± 0.43
(mmol/L)		
2-h plasma glucose (2hPG)		5.88 ± 1.42
(mmol/L)		
GDM according to MOH criteria^e^	48 (10.6)	
GDM according to IADPSG criteria^f^	57 (12.6)	

a *1 USD = RM 4.18*.

b *Data expressed as medians, p (25, 75)*.

c *Recommended Nutrient intakes (RNI) for Malaysia*.

d *RNI for Malaysia: pre-pregnancy (19–29 years old−53 g/day, 30–59 years old−52 g/day); first trimester (19–29 years old−53.5 g/day, 30–59 years old−52.5 g/day); second trimester (19–29 years old−61 g/day, 30–59 years old−60 g/day) (30)*.

e *Gestational diabetes mellitus (GDM) according to the Ministry of Health (MoH) criteria, either of both fasting plasma glucose (FPG) ≥ 5.6 mmol/L or 2-h plasma glucose (2hPG) ≥ 7.8 mmol/L*.

f *GDM according to IADPSG criteria, either of both FPG ≥ 5.1 mmol/L or 2hPG ≥ 8.5 mmol/L*.

Table 2 shows the types and sources of protein intake before and during pregnancy. Overall, the women significantly increased or reduced their total protein intake (pre-pregnancy = 59.43 g/day, first trimester = 55.84 g/day, second trimester = 59.30 g/day, and *p* = 0.02) and plant protein (pre-pregnancy = 23.37 g/day, first trimester = 21.93 g/day, second trimester = 22.07 g/day, and *p* = 0.04), but not animal protein from pre-pregnancy to the second trimester (pre-pregnancy = 34.66 g/day, first trimester = 33.46 g/day, second trimester = 35.34 g/day, and *p* > 0.05). For animal protein source, there were significant differences in the median protein intake from fish, seafood, and milk across pregnancy trimesters. The intakes of protein from fish and seafood were highest before pregnancy but lowest in the first trimester. An increase in milk protein intake was observed during pregnancy from pre-pregnancy to first trimester until second trimester. Protein intake from the other sources did not show any significant difference across the pregnancy trimesters.

**Table 2:** The types and sources of protein intake before and during pregnancy.

**Source of protein**	**Pre-pregnancy**	**First trimester** **(Week 10–13)**	**Second trimester** **(Week 24–30)**	***p*** **-value**
	**Median** ***p*** **(25, 75) (g/day)**			
Total protein	59.43 (48.02, 78.22)^a^	55.84 (42.30, 76.17)^a^	59.30 (45.04, 76.17)	0.02^*^
Animal protein	34.66 (25.97, 47.71)	33.46 (23.47, 45.79)	35.34 (25.90, 46.65)	0.17
Poultry	4.80 (3.24, 11.16)	4.84 (3.24, 11.90)	4.80 (3.24, 9.60)	0.10
Red meat	2.04 (0.84, 3.98)	1.72 (0.45, 3.83)	1.90 (0.62, 4.40)	0.06
Processed meat	1.33 (0.36, 3.14)	1.04 (0.25, 2.64)	1.15 (0.30, 2.49)	0.10
Fish	10.72 (6.02, 16.93)^a^	9.63 (5.18, 16.28)^a^	10.05 (6.00, 16.52)	0.04^*^
Seafood	1.05 (0.17, 2.26)^a^	0.66 (0.04, 1.57)^a^,^c^	0.84 (0.18, 2.11)^c^	0.01^*^
Eggs	2.75 (1.36, 4.21)	2.10 (0.95, 3.94)	2.85 (1.27, 3.94)	0.14
Milk	1.58 (0.13, 5.72)^a^, ^b^	3.85 (0.23, 8.95)^b^, ^c^	5.93 (2.59, 9.96)^a^, ^c^	0.001^**^
Dairy products	0.82 (0.36, 1.78)	0.72 (0.18, 2.12)	0.72 (0.18, 2.12)	0.65
Plant protein	23.37 (16.90, 32.16)^a^	21.93 (15.88, 29.40)^a^	22.07 (16.82, 29.31)	0.04^*^
Nuts, seeds and legume	1.39 (0.34, 3.51)	1.89 (0.60, 4.21)	1.72 (0.76, 3.26)	0.06
Vegetables	1.55 (0.59, 3.47)	2.24 (1.02, 4.12)	1.63 (0.88, 2.92)	0.05
Fruits	1.00 (0.55, 1.97)	1.34 (0.64, 2.58)	1.40 (0.71, 2.57)	0.10
Grains, cereal and cereal products	16.24 (12.12, 21.85)	14.90 (9.29, 19.46)	15.44 (12.18, 21.01)	0.06

*RNI for Malaysia: pre-pregnancy (19–29 years old−53 g/day, 30–59 years old−52 g/day); first trimester (19–29 years old−53.5 g/day, 30–59 years old−52.5 g/day); second trimester (19–29 years old−61 g/day, 30–59 years old−60 g/day) (30)*.

*The medians with similar superscripts in the same row indicate a significant difference (p < 0.05):*

a *pre-pregnancy vs. first trimester;*

b *pre-pregnancy vs. second trimester;*

c *first trimester vs. second trimester*.

* *p < 0.05*,

** *p < 0.001*.

Table 3 shows the associations between protein intake (total protein, animal protein, and plant protein) with the risk of GDM. No significant associations were observed between the total plant protein intake and source of plant protein intake with the risk of GDM. The women in the highest tertile (T3) of animal protein intake in the second trimester were significantly associated with the risk of GDM (AOR = 2.76, 95% *CI* = 1.27–6.04) compared with the women in the middle tertile of intake (T2). Further analysis showed that the women in the T3 of red meat protein in the second trimester had significantly higher risk for GDM (AOR = 2.69, 95% *CI* = 1.27–5.70) compared with the women in the T2 of red meat protein (Table 4). On the contrary, the women in the T3 of egg protein in the second trimester were significantly at lower risk of GDM (AOR = 0.43, 95% *CI* = 0.18–0.91) compared with those in T2.

**Table 3:** Adjusted odds ratio (AOR) and 95% *CI* for associations between the total and types of protein intake with GDM.

	**Intake tertile** ^**a**^		
	**T1**	**T2**	**T3**
	**AOR [95% CI]**		
Total protein (g/day)			
Pre-pregnancy	0.89 [0.40–1.98]	1.00	1.26 [0.58–2.77]
First trimester	0.89 [0.40–1.97]	1.00	1.68 [0.75–3.77]
Second trimester	0.87 [0.41–1.83]	1.00	1.33 [0.58–3.01]
Animal protein^b^ (g/day)			
Pre-pregnancy	0.73 [0.33–1.63]	1.00	1.18 [0.55–2.56]
First trimester	0.75 [0.35–1.62]	1.00	0.98 [0.44–2.19]
Second trimester	0.67 [0.27–1.64]	1.00	2.76 [1.27–6.04]^*^
Plant protein (g/day)			
Pre-pregnancy	0.95 [0.43–2.09]	1.00	0.88 [0.41–1.87]
First trimester	1.65 [0.78–3.48]	1.00	0.72 [0.32–1.61]
Second trimester	1.41 [0.66–3.10]	1.00	0.78 [0.56–2.69]

*RNI for Malaysia: pre-pregnancy (19–29 years old−53 g/day, 30–59 years old−52 g/day); first trimester (19–29 years old−53.5 g/day, 30–59 years old−52.5 g/day); second trimester (19–29 years old−61 g/day, 30–59 years old−60 g/day) (30)*.

a *Pre-pregnancy: Total protein (T1 = ≤ 51.45, T2 = 51.46–70.41, T3 = ≥ 70.42), Animal protein (T1 = ≤ 28.67, T2 = 28.68–43.48, T3 = ≥ 43.49), Plant protein (T1= ≤ 18.93, T2 = 18.94–28.36, T3 = ≥ 28.37)*. *First trimester: Total protein (T1 = ≤ 46.39, T2 = 46.40–66.66, T3 = ≥ 66.67), Animal protein (T1 = ≤ 26.32, T2 = 26.33–41.10, T3 = ≥ 41.11), Plant protein (T1 = ≤ 17.89, T2 = 17.90–25.44, T3 = ≥ 25.45)*. *Second trimester: Total protein (T1 = ≤ 50.52, T2 = 50.53–70.51, T3 = ≥ 70.52), Animal protein (T1 = ≤ 28.93, T2 = 28.94–42.14, T3 = ≥ 42.15), Plant protein (T1 = ≤ 18.88, T2 = 18.89–26.46, T3 = ≥ 26.47)*.

b *Animal protein included poultry, red meat, processed meat, fish, seafood, eggs, milk, and dairy products*. *Adjusted by age, parity, family history of diabetes mellitus (DM), history of GDM, pre-pregnancy body-mass index (BMI), energy intake, and fat intake*.

* *p < 0.05*.

**Table 4:** Adjusted odds ratio (AOR) and 95% *CI* for the associations between the animal protein sources and GDM.

**Source of animal protein**	**Intake tertile**		
	**T1**	**T2**	**T3**
	**AOR [95% CI]**		
Red meat			
Pre-pregnancy	1.45 [0.64–3.28]	1.00	1.66 [0.76–3.63]
First trimester	1.02 [0.42–2.48]	1.00	2.11 [0.99–4.42]
Second trimester	0.61 [0.25–1.50]	1.00	2.69 [1.27–5.70]^**^
Processed meat			
Pre-pregnancy	0.60 [0.26–1.37]	1.00	1.18 [0.56–2.52]
First trimester	1.53 [0.65–3.63]	1.00	1.80 [0.83–3.88]
Second trimester	1.31 [0.56–3.08]	1.00	1.43 [0.64–3.23]
Poultry			
Pre-pregnancy	0.77 [0.36–1.62]	1.00	0.94 [0.43–2.03]
First trimester	1.49 [0.66–3.35]	1.00	1.10 [0.45–2.67]
Second trimester	0.95 [0.48–2.07]	1.00	1.01 [0.43–2.20]
Fish			
Pre-pregnancy	0.85 [0.40–1.80]	1.00	0.69 [0.32–1.48]
First trimester	0.79 [0.36–1.72]	1.00	1.19 [0.57–2.51]
Second trimester	1.40 [0.64–3.04]	1.00	1.50 [0.67–3.32]
Seafood			
Pre-pregnancy	1.04 [0.49–2.21]	1.00	1.10 [0.50–2.41]
First trimester	1.02 [0.46–2.29]	1.00	1.68 [0.78–3.67]
Second trimester	0.83 [0.39–1.78]	1.00	1.09 [0.51–2.36]
Eggs			
Pre-pregnancy	1.17 [0.57–2.41]	1.00	0.78 [0.31–1.98]
First trimester	1.02 [0.49–2.13]	1.00	1.09 [0.49–2.40]
Second trimester	0.83 [0.40–1.70]	1.00	0.43 [0.18–0.91]^*^
Milk			
Pre-pregnancy	0.52 [0.24–1.13]	1.00	1.02 [0.49–2.13]
First trimester	1.16 [0.53–2.53]	1.00	1.47 [0.67–3.26]
Second trimester	0.74 [0.37–1.51]	1.00	1.18 [0.62–2.22]
Dairy products			
Pre-pregnancy	1.50 [0.64–3.49]	1.00	2.49 [0.99–6.03]
First trimester	0.73 [0.35–1.53]	1.00	0.90 [0.42–1.96]
Second trimester	0.98 [0.48–2.01]	1.00	0.76 [0.34–1.73]

^a^*Pre-pregnancy: red meat (T1 = ≤ 1.32, T2 = 1.33–3.39, T3 = ≥ 3.40), processed meat (T1 = ≤ 0.66, T2 = 0.67–2.10, T3 = ≥ 2.11), poultry (T1 = ≤ 3.24, T2 = 3.25–9.60, T3 = ≥ 9.61), fish (T1 = ≤ 8.39, T2 = 8.40–15.12, T3 = ≥ 15.13), seafood (T1 = ≤ 0.33, T2 = 0.34–1.67, T3 = ≥ 1.68), eggs (T1 = ≤ 1.90, T2 = 1.91–3.93, T3 = ≥ 3.94), milk (T1 = ≤ 0.63, T2 = 0.64–3.85, T3 = ≥ 3.86), dairy products (T1 = ≤ 0.54, T2 = 0.55–1.26, T3 = ≥ 1.27)*.

*First trimester: red meat (T1 = ≤ 0.93, T2 = 0.94–2.78, T3 = ≥ 2.79), processed meat (T1 = ≤ 0.46, T2 = 0.47–1.73, T3 = ≥ 1.74), poultry (T1 = ≤ 3.24, T2 = 3.25–6.36, T3 = ≥ 6.37), fish (T1 = ≤ 6.09, T2 = 6.10–13.01, T3 = ≥ 13.02), seafood (T1 = ≤ 0.31, T2 = 0.32–1.27, T3 = ≥ 1.28), eggs (T1 = ≤ 1.55, T2 = 1.56–3.39, T3 = ≥ 3.40), milk (T1 = ≤ 1.25, T2 = 1.26–7.64, T3 = ≥ 7.65), dairy products (T1 = ≤ 0.36, T2 = 0.37–1.48, T3 = ≥ 1.49)*.

*Second trimester: red meat (T1 = ≤ 0.93, T2 = 0.94–2.93, T3 = ≥ 2.94), processed meat (T1 = ≤ 0.38, T2 = 0.39–1.83, T3 = ≥ 1.84), poultry (T1 = ≤ 3.24, T2 =, 3.25–6.36 T3 = ≥ 6.37), fish (T1 = ≤ 7.89, T2 = 7.90–12.75, T3= ≥ 12.76), seafood (T1 = ≤ 0.34, T2 = 0.35–1.36, T3 = ≥ 1.37), eggs (T1 = ≤ 1.90, T2 = 1.91–2.85, T3 = ≥ 2.86), milk (T1 = ≤ 3.85, T2 = 3.86–8.95, T3 = ≥ 8.96), dairy products (T1 = ≤ 0.36, T2 = 0.37–1.33, T3 = ≥ 1.34)*.

*Adequate GDM as reference group*.

*Adjusted by age, parity, family history of DM, history of GDM, pre-pregnancy BMI, energy intake, and fat intake*.

* *p < 0.05*

** *p < 0.001*.

## Discussion

This study demonstrates that the highest tertile of animal protein intake (≥42.15 g/day) in the second trimester, but not at pre-pregnancy or in early pregnancy, was significantly associated with the risk of GDM. This finding was consistent with the previous studies in that dietary intake during mid-pregnancy was associated with GDM the risk (8, 37–40). During the first trimester of pregnancy, almost all the women experience loss of appetite, nausea, and vomiting, making it difficult to obtain sufficient nutrition (41). In a normal physiological process of pregnancy, an ~50–70% reduction of maternal insulin sensitivity occurs in mid-pregnancy so that the nutrients are shunted to the growing fetus (42). A pregnant woman may develop raised blood glucose concentration (hyperglycemia) or GDM if her pancreatic beta-cells are unable to increase insulin secretion and overcome insulin resistance. Thus, maternal nutrition in the second trimester can be considered as an important turning point for both the mother and fetus.

The present study found that the highest tertile of red meat protein (≥ 2.94 g protein per day or equivalent to roughly 12 g of cooked red meat per day) in the second trimester was significantly associated with the risk of developing GDM. The previous studies have shown inconsistent results, with the two studies suggesting significant associations (15, 43) and one study demonstrated a non-significant association (8). However, direct comparisons between the studies could not be made because different measurements for meat intake were used. A cohort study in China showed that the women with the highest meat intake (pork, beef, chicken, or lamb) had a higher risk for GDM than those in the lowest meat intake tertile (15). Similarly, a prospective study on the association between the food habits and the GDM risk in India showed that eating red meat more than three times per week was significantly associated with the GDM risk after adjusted for the covariates (43). In contrast, a cohort study in Singapore (Growing Up in Singapore Toward healthy Outcomes—GUSTO study) showed that higher protein from red meat was not significantly associated with a higher risk of GDM (8). The low red meat intake (median = 24.38 g/day, 25 and 75th percentiles = 26.90 and 38.43 g/day, respectively) reported in this study may not explain that the observed association between red meat intake and GDM risk is due to the red meat components, such as saturated fat and cholesterol. Rather, the association could be attributed to the common cooking methods of red meat and the overall animal protein intake of the women. Fried red meat cooked with chili/soy sauce/ketchup or mixed in fried vegetables, red meat cooked in coconut milk-based dishes, and grilled marinated red meat with spices (satay) served with ground-nut gravy are among common food preparation of red meat in the Malaysian food culture. These cooking methods not only contribute to a higher overall fat and energy content of the diet but also increase the production of several hazardous chemicals, such as heterocyclic amines (HCAs), polycyclic aromatic hydrocarbons (PAHs), and advanced glycation end products, which are known carcinogens that can decrease insulin sensitivity and further contribute to GDM development (44, 45). Additionally, the present study found that the women in the highest tertile of red meat intake had significantly higher mean animal protein intake compared with the women in the lowest tertile of red meat intake (47.94 vs. 33.30 g/day of animal protein, *p* < 0.001). As the precise mechanism remains unclear for now, it might be that the overall animal protein intake or the carcinogens produced by the red meat cooking methods contribute to the GDM risk (44).

It has been reported that the higher egg consumption (>4 eggs/week) increased the risk of T2DM (46, 47) and GDM (48). However, the present study found an inverse association between egg intake (≥ 2.86 g protein per day or equivalent to approximately half of an egg per day or 3–4 eggs/week) and GDM risk. The comparison between the studies should be done with caution, owing to the different time period measured as well as the number of eggs consumed in these studies. Qin et al. (48) reported that the women with high pre-pregnancy and early-pregnancy egg consumption of seven or more eggs per week had 1.8 times increased risk of GDM as compared with the women consuming fewer than seven eggs per week (adjusted relative risk (ARR) = 1.77, 95% *CI* = 1.19–2.63). The present study found that higher egg intake in the second trimester, but not at pre-pregnancy or in early pregnancy, was significantly associated with lower risk of GDM. The median egg intake for the women in the second trimester was 0.65 (0.50, 1.03) serving/day (~ half of an egg). The high-quality protein from egg and other components in eggs, such as unsaturated fats and lecithin, could be the possible explanation for the inverse association between the egg intake and GDM. The studies showed that omega-3 polyunsaturated fatty acids, mainly docosahexaenoic acid (DHA) in the eggs, have been associated with chronic diseases, such as metabolic syndrome, DM, and hypercholesterolemia, as well as the risk of death from any cause-specific mortality (49, 50). It is also important to note that the studies conducted in the Asian populations also found that egg consumption (~3– < 6 eggs/week) was significantly associated with the lower risk of CVD (e.g., stroke and coronary heart disease) (51, 52). Taken all together, these findings suggest that egg consumption in moderate amount (1–3 eggs/week) may exert beneficial health effects among the Asian populations. However, the protective role of egg consumption in improving the pregnancy outcomes needs to be further confirmed by more studies with larger sample size.

A plant-based protein diet is known to be protective against obesity, NCD, T2DM, and GDM as well as all-cause mortality (9, 53, 54). Plant protein contains plenty of antioxidants and rich in dietary fiber, which may lessen the negative effects of inflammation, insulin resistance, as well as oxidative damage (55). However, several studies reported that plant protein was not significantly associated with the risk of T2DM (10) and GDM (15). Similarly, the present study found that the higher intake of plant protein was inversely associated with GDM risk, but this association was not statistically significant. The health benefits of plant proteins or as alternative to animal protein have yet to be investigated extensively. Although the results are not conclusive, in general, a well-planned plant-based diet that includes the vegetables, fruits, whole grains, legumes, nuts, and seeds can provide balanced nutrition to meet the individual needs and prevent the nutrient deficiencies (56). Thus, the challenge should be how to encourage the pregnant women to increase their plant protein intake.

The higher consumption of dairy products is associated with the reduced risks of obesity, T2DM, and GDM (57–59), and this favorable association depends in the type of dairy products (e.g., low-fat or high-fat) and the amount of dairy products consumed. However, the present study showed that protein from the dairy products was not significantly associated with GDM risk. In contrast, the GUSTO study showed that higher dairy protein (AOR = 1.87; 95% *CI* = 1.11–3.15) were significantly associated with a higher GDM risk. This non-significant association could be due to the generally low intake of dairy product as 96.4% of the women had ≤ 1 serving/day. The reasons for the low level of dairy products intake are likely due to the multiple causes, such as preference of taste, eating habits, and family environment. Thus, the strategies to increase the dairy products intake to the optimal levels are needed.

This study is not without limitations. As pre-pregnancy weight and dietary intake during pregnancy were self-reported, the GWG and diet data might have been subject to misclassification error. However, the self-reported maternal weight data has been shown to provide the reliable estimates (60). Albums of foods and beverages and household measurements were used to assist the recall of dietary intake of respondents to prevent the errors in reporting or recalling portion sizes of the consumed foods. The cut-off point for protein intake for each protein source is not standardized, whereby it was divided by the tertiles based on the distribution of intakes. The use of the USDA food database, rather than the local food composition database to estimate the protein content of foods might result in overestimation of the reported values. However, it is not expected that there is much difference in the protein content of foods in the local food composition database with those in the USDA food database. Finally, this study did not consider the overall diet quality, although the multivariate analyses did adjust for energy intake that could proxy the quality of diet, e.g., higher energy-dense diet may be of lower nutrient-dense. In addition, there are several strengths to this study, such as the prospective study design, and the repeated assessments of protein intake at various periods of pregnancy, from pre-pregnancy through the second trimester of pregnancy.

In conclusion, protein intake from the animal sources in the second trimester was associated with an increased risk of GDM. Most of the women in this study had adequate protein intake before and during pregnancy. Thus, limiting red meat intake and replacement with the lean sources of protein, such as fish, poultry, and eggs could be beneficial in the prevention of GDM. These findings support that not only the quantity of protein intake during pregnancy is important, but also the quality of dietary protein should be considered. More studies on the associations between risk of GDM with the types and sources of protein in the context of overall diet, such as the specific mechanisms underlying such associations, are warranted.

## Data Availability Statement

The raw data supporting the conclusions of this article will be made available by the authors, without undue reservation.

## Ethics Statement

The studies involving human participants were reviewed and approved by the Medical Research Ethics Committee (MREC), Universiti Putra Malaysia (UPM/FPSK/100-9/2-MJKEtika), and the Medical Research Ethics Committee (MREC), Ministry of Health Malaysia (KKM/NIHSEC/08/0804/P12-613). The patients/participants provided their written informed consent to participate in this study.

## Author Contributions

ZM is the project leader of the SECOST study and designed the project with HY. HY conducted the literature search, data collection, statistical analyses, and wrote the first draft of the paper. BM, ZR, YT, and JB contributed to methodology and resources. ZM and EB revised the subsequent drafts for important intellectual content and approved the final version of the paper to be published. All authors read and approved the final manuscript.

## Funding

This work was supported by the Danone Dumex (Malaysia) Sdn Bhd [6368500].

## Conflict of Interest

The authors declare that this study received funding from Danone Dumex (Malaysia). The funder had the following involvement in the study: methodology, interpretation of data and review of manuscript.

## Publisher's Note

All claims expressed in this article are solely those of the authors and do not necessarily represent those of their affiliated organizations, or those of the publisher, the editors and the reviewers. Any product that may be evaluated in this article, or claim that may be made by its manufacturer, is not guaranteed or endorsed by the publisher.
