# Supplementary material for: Higher Animal Protein Intake During the Second Trimester of Pregnancy Is Associated With Risk of GDM
Source: Front Nutr. 2021 Nov 15;8:718792. doi: 10.3389/fnut.2021.718792 (PMC8634888; doi:10.3389/fnut.2021.718792)
Supplement: Supplementary file 1 [file Table_1.DOCX]

**Supplementary Table 1: Food groups and food items**

| **Food groups** | **Food items** |
| --- | --- |
| Poultry | Chicken, duck, goose, turkey, quail, |
| Red meat | Beef, lamb/goat, buffalo, pork, rabbit, organs and glands such as liver, kidney, brain and heart |
| Processed meat | Burger, sausage, ham, nugget, chicken/seafood/fish balls |
| Fish | Marine fish, freshwater fish, anchovy, canned fish, dried fish, fish snacks |
| Seafood | Mussels, oysters, scallops, cockles/clams, shrimp, prawn, cuttlefish/squid, lobster, crab |
| Eggs | Chicken eggs, duck eggs, quail eggs, salted eggs |
| Milk | Fresh milk, flavored milk, ultra-heat-treated milk, powdered milk |
| Dairy products | Yogurt, ice-cream, cheese, cultured milk |
| Nuts, seeds & legumes | Nuts/pulses, peanuts, dried seeds, almonds, cashews, walnuts, peas, baked beans, kidney beans, lentils (red, green, yellow and brown), dal, soybean curd, fermented soy |
| Vegetable | All types, e.g. French beans, angled beans |
| Fruits | Guava, melon, local orange/mandarin orange, mango, pineapple, banana, watermelon, star fruit, apple, orange, pear, grape, longan, honeydew |
| Grains, cereal & cereal products | White rice, brown rice, glutinous rice, porridge, wheat noodle, rice noodles, pasta, spaghetti, bread, buns, pastries, roti canai, thosai, chapati, pizza, bagels, oats, corn, barley, sago, breakfast cereals, instant cereals |
